# Complete mitochondrial genome of white-throated rock-thrush *Monticola cinclorhynchus gularis* (Passeriformes: muscicapidae)

**DOI:** 10.1080/23802359.2016.1219640

**Published:** 2016-09-01

**Authors:** Hao Zhang, Yuanyuan Cheng, Lizhi Zhou, Yuanqiu Dong

**Affiliations:** a School of Resources and Environmental Engineering, Anhui University, Hefei, P. R. China;; b Anhui Biodiversity Information Center, Hefei,P. R. China

**Keywords:** *Monticola cinclorhynchus gularis*, white-throated rock-thrush, complete mitochondrial genome, gene arrangement

## Abstract

The white-throated rock-thrush *Monticola cinclorhynchus gularis* is a subspecies bird in the family Muscicapidae of the order Passeriformes, which is distributed in Southeast Siberia to Northeast China and Korea, winters to Southeast Asia. In this study, we determined its complete mitochondrial genome (mtDNA) by the PCR-based method. The complete mtDNA is a 16,801 bp circular molecule containing 37 typical genes. The gene order is identical with the standard avian gene order. All protein-coding genes start with a typical ATG codon except for COI. The termination codon is usually the standard TAA. The 12S rRNA is 984 bp, and the 16S rRNA is 1602 bp in length. All tRNAs possess the classic clover leaf secondary structure except for tRNA^Ser (AGN)^ and tRNA^Cys (CUN)^, which lack the ‘DHU’ stem, only forming a simple loop. The non-coding regions contain a 1222 bp long control region (D-loop) and a few intergenic spacers. The phylogenetic tree based on some mitogenomes from Muscicapidae and Turdidae indicates that *M. cinclorhynchus gularis* and *Phoenicurus auroreus* are in the same branch in the group Muscicapidae.

The white-throated rock-thrush *Monticola cinclorhynchus gularis* is a subspecies bird in the family Muscicapidae of the order Passeriformes, which distributes in Southeast Siberia to Northeast China and Korea, winters to Southeast Asia. *M. c. gularis* has usually been identified as Turdinae bird species in Muscicapidae (Cheng 1987) in traditional taxonomy, however, some scholars think it should be seen as Muscicapidae bird and was split into two species recently, one of its subspecies *M. c. gularis* gave rise to a new species *M. gularis* (Dickinson 2003), or as Turdidae bird (Zheng 2011). In this study, we sequenced the complete mitochondrial genome of the white-throated rock-thrush *M. c. gularis*, in order to provide basic data for the systematic study of this species.

The whole genomic DNA was isolated from alcohol-preserved muscle tissues using traditional phenol/chloroform method (Hughes & Baker 1999; Liu et al. 2013), and later the complete mitogenome was amplified by PCR method. The samples were collected from an individual dead by accident which hit glass in the Qingyuan village of Anhui university, Hefei, China, on 13 May 2015. It was kept in the Institute of Biodiversity and Wetland Ecology, Anhui University, China (Voucher MC20150513).

The complete mitochondrial genome DNA of *M. c. gularis* is 16,801bp in length (GenBank accession no. KX506858). It has a typical circular mitochondrial genome, which contains 13 protein-coding genes, 2 rRNA genes, 22 putative tRNAs, and a non-coding A + T-rich region. The overall base composition (H-strand) is A, 29.74%; C, 32.33%; G, 14.52%, and T, 23.41%. A + T content is 53.15%, within the range for avian mitogenomes (51.6–55.7%; Haring et al. 2001). The order and orientation are identical with the standard avian gene order (Gibb et al. 2007). All protein-coding genes utilize the standard start codon ATG, except for the *COI* gene starting with GTG. TAA is the most frequent stop codon, although *ND1* and *ND*5 end with AGA, *ND6* ends with TAG, and *COI* ends with AGG, *COIII* and *ND4* stop with the single nucleotide T––. The 12S rRNA is 984 bp, and the 16S rRNA is 1602 bp in length, which is located between tRNA^Phe^ and tRNA^Leu^, and separated by tRNA^Val^. Most of the mitochondrial genes are encoded on heavy strand (H-strand) while ND6 and eight tRNA genes are encoded on light strand (L-strand). All tRNAs possess the classic clover leaf secondary structure except for tRNA^Ser (AGN)^ and tRNA^Cys (CUN)^, which lack the ‘DHU’ stem, only forming a simple loop, as observed in other bird mitogenomes (Bernt et al. 2012). The non-coding regions contain a 1222 bp long control region (D-loop), which is located between tRNA^Glu^ and tRNA^Phe^, and a few intergenic spacers. In addition, 85 bp intergenic spacer sequences are spread over 16 regions, 12 areas of gene overlap occupying a total of 33 bp throughout the whole genome.

In order to understand the phylogenetic status of white-throated rock-thrush, we reconstructed phylogenetic trees based on our mitogenome and the data from GenBank using the Bayesian inference (BI) and neighbour-joining (NJ) methods, with *Gallus gallus* as an outgroup. The phylogenetic trees share similar topologies and high-node support values (Figure 1). It appears that Muscicapidae and Turdidae are sister groups, these two families have close relationship. The phylogenetic tree indicates *M. c. gularis* and *Phoenicurus auroreus* are in the same branch in the group Muscicapidae.

**Figure 1.**
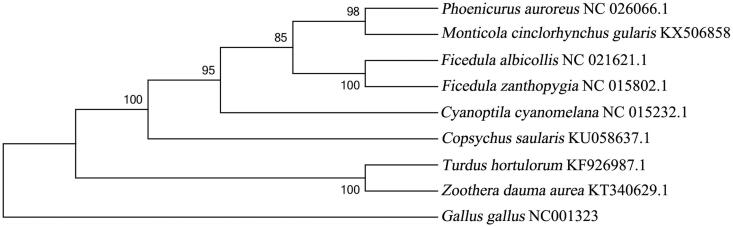
The phylogenetic tree of nine species Passeriformes birds constructed using the neighbour-joining method based on complete mtDNA sequences, and *Gallus gallus* (NC001323) was used as an outgroup. The tree was built using the Kimura-two-parameter (K2P) model, and the numbers on the branches are bootstrap values. The eight species Passeriformes contain six Muscicapidae birds: *Cyanoptila cyanomelana* (HQ896033.1), *Ficedula zanthopygia* (JN018411.1), *F. albicollis* (KF293721.1), *Phoenicurus auroreus *(KF997863.1), *Copsychus saularis* (KU058637.1), *Monticola cinclorhynchus gularis* (KX506858), and two Turdidae birds: *Turdus hortulorum* (KF926987.1) and *Zoothera dauma aurea* (KT340629.1).
